# The effects of compensatory workplace exercises to reduce work-related stress and musculoskeletal pain^1^

**DOI:** 10.1590/0104-1169.3222.2461

**Published:** 2014

**Authors:** Fabiana Cristina Taubert de Freitas-Swerts, Maria Lúcia do Carmo Cruz Robazzi

**Affiliations:** 2 PhD; 3 PhD, Full Professor, Escola de Enfermagem de Ribeirão Preto, Universidade de São Paulo, WHO Collaborating Centre for Nursing Research Development, Ribeirão Preto, SP, Brazil

**Keywords:** Exercise Therapy, Burnout, Professional, Musculoskeletal Pain, Physical Therapy Modalities, Occupational Health, Worker

## Abstract

**OBJECTIVES:**

to assess the effect of a compensatory workplace exercise program on workers with the purpose of reducing work-related stress and musculoskeletal pain.

**METHOD:**

quasi-experimental research with quantitative analysis of the data, involving 30 administrative workers from a Higher Education Public Institution. For data collection, questionnaires were used to characterize the workers, as well as the Workplace Stress Scale and the Corlett Diagram. The research took place in three stages: first: pre-test with the application of the questionnaires to the subjects; second: Workplace Exercise taking place twice a week, for 15 minutes, during a period of 10 weeks; third: post-test in which the subjects answered the questionnaires again. For data analysis, the descriptive statistics and non-parametric statistics were used through the Wilcoxon Test.

**RESULTS:**

work-related stress was present in the assessed workers, but there was no statistically significant reduction in the scores after undergoing Workplace Exercise. However, there was a statistically significant pain reduction in the neck, cervical, upper, middle and lower back, right thigh, left leg, right ankle and feet.

**CONCLUSION:**

the Workplace Exercise promoted a significant pain reduction in the spine, but did not result in a significant reduction in the levels of work-related stress.

## Introduction

Problems concerning work-related stress are associated with globalization, increase of the informal economy and changes that occur in the workplace. Organizations usually consider as preventive aspects in occupational health and safety the care taken in relation to the exposure to chemical, physical and biological agents and are not concerned with the psychosocial risk factors, which are overlooked and poorly understood because they are difficult to be measured and identified as objectively as the other environmental risks^(^ 1 ^-^ 2 ^)^. 

Psychosocial factors involve subjective symptoms such as physical or mental exhaustion, fatigue and stress, overload, time pressure and low control over the work^(^ 2 ^)^, besides significantly contributing to the incidence and severity of Work-related Musculoskeletal Disorders (WRMD). Muscular tension caused by stress may occur partially by the relationship between psychosocial factors and musculoskeletal disorders, due to the close relationship between psychosocial, biomechanical, organizational and individual variables in the development and intensification of this multifactorial situation^(^ 3 ^-^ 5 ^)^.

Therefore, the psychosocial factors at the workplace are involved in the etiology of the WRMD, especially in the context of office work involving computer stations. However, the importance of physical ergonomic factors and biomechanical mechanisms found in the etiology of these disorders should not be diminished, but a more holistic view of this situation that involves physical and ergonomics components, as well as psychosocial ones, should preferably be taken^(^ 1 ^-^ 5 ^)^.

Brazilian companies spend an excessive amount of money with accidents, work-related illnesses and stress. These costs highlight the need for prevention programs covering multiple causal and relevant factors concerning work-related stress and illnesses^(^ 2 ^)^. These measures are aimed at reducing the exposure to these factors, in a diversified and multidisciplinary manner, with the implementation of a Workplace Exercise (WE) program as one of the possible ways to be adopted^(^ 6 ^-^ 8 ^)^.

Commonly known as workplace gym, the WE is a prevention and compensatory activity considered as one of the measures used to cope with physical and emotional disorders, and is aimed at preventing the illnesses that can result from repetitive and boring work and that can lead to workplace accidents and low productivity^(^ 6 ^-^ 7 ^)^. Its use is important as one of the preventive initiatives proposed by the different professionals working in the occupational health field. For such, it should be well planned and diversified, since it consists of an active break at work, characterized by an exercise program, static and dynamic stretching and muscular strengthening and it is intended to break the continuity of the task performed by the worker^(^ 6 ^-^ 8 ^)^. 

It is known that the WE alone can bring benefits, but these are greater when this activity takes place in conjunction with others for the promotion of workers' health. A Health Promotion Program in a Brazilian oil company took place during the year of 2008 and it involved physical educators, physiotherapists, doctors, nurses, social assistants and psychologists. The initiative took place throughout that year, which included: immunization, hygienic food control and water treatment for domestic use procedures and exercises practiced daily in the workplace, among others. The Program helped build a culture of health promotion and the study showed that well informed workers are healthier, more productive and possibly happier^(^ 9 ^)^.

A study carried out in Thailand involving nurses was aimed at assessing the effects of physical exercise on physical fitness related to the health of these workers. Regular physical exercise, including working out, has a positive effect on the physical status. The study was quasi-experimental and carried out in a medical center, where 95 nurses of different units of a hospital volunteered to participate in a three-month intervention program. Indicators of physical fitness related to the health of both groups were established and assessed before and after the intervention. The study showed that the development and implementation of an intervention program may support and improve physical fitness related to the health of nurses. These workers should be engaged in exercise programs, at their workplace, to reduce the risk of musculoskeletal injuries and enhance their efficiency^(^ 10 ^)^.

The effects of the physical exercises intervention at the workplace in relation to subjective wellbeing, psychosocial and physical functioning, as well as general wellbeing were investigated in Finland. The study was controlled randomized and the subjects were office workers. The variables psychosocial functioning and wellbeing were measured by visual scales. The *design* of the type *cross-over* consisted of a period of light resistance training and guidance of 15 weeks and another period without any training and guidance. The statistical analysis was based on linear mixed models. The active component of the intervention, the light resistance training, resulted in a statistically significant increase in subjective physical wellbeing (p=0.015). The physical exercise intervention had no effect over the somatic symptoms, anxiety, self-confidence, mood, work-related stress, workplace environment, life satisfaction or life meaning. The light resistance training performed during the working day had a positive effect on the subjective physical wellbeing among the assessed workers^(^ 11 ^)^.

The present study supports the progress of the multidisciplinary knowledge related to workers' health, taking into account the gap in the production of national knowledge^(^ 6 ^)^ concerning Workplace Exercise proposed herein.

Given the above, this study was aimed at assessing the effect of a compensatory Workplace Exercise program applied to administrative staff working in a public educational institution, with the purpose of reducing the complaints associated with work-related stress and musculoskeletal pain.

## Method

Research of quasi-experimental design, with quantitative and comparative analysis of data, developed in a Higher Education Public Institution (HEPI) which provides education to healthcare professionals and is located in Ribeirao Preto, Sao Paulo state.

The study population was composed of 67 employees who were allocated to administrative areas. Of these, 45 confirmed their participation in the WE, but only 30 fully complied with the proposed activity and this was the final subject sample. The inclusion criteria adopted were: workers with minimum of one year working at the institution, who did not have any physical or mental issues that, through medical advice, could prevent them from participating in the activity during data collection, and who worked in the administrative areas of the HEPI, with the purpose of ensuring that the working activities and tasks performed by the subjects were similar.

Those who were on sick leave, maternity leave, pregnant women, physically disabled and those undertaking physiotherapy and psychotherapy due to pain and stress were excluded. In addition to the exclusion criteria, some of the reasons for employees not to participate were: not having the agreement of immediate managers to perform the WE; accumulation or excess of duties in the department in which they worked, preventing them to continue participating; illnesses occurred in the course of the period set for data collection; lack of time for its performance, among others.

Research carried out in a Brazilian telemarketing center also showed low adherence to the WE program, despite its undeniable gains when workers are allowed to be aware of their bodies; however, the awareness about the body goes through a learning process, in which some beliefs and behaviors are revisited in order to be destroyed. It seems that the work does not allow and sometimes prevent the adherence to the WE program, even though this is formally encouraged by the organizers of this production^(^ 12 ^)^.

In the present investigation, a pilot study was conducted prior to data collection, with a sample of 11 subjects of different administrative sectors of the institution. Based on this process, it was possible to refine the method of research, improving the dynamics of data collection and pre-establishing the manner in which the statistical analysis would be conducted. It was also possible to assess the choice of data collection instruments used and their effectiveness both in the application and understanding of the subjects concerning the easiness in obtaining answers. In relation to the WE, a protocol previously tested was used^(^ 8 ^)^, which was changed with the addition of segmental stabilization techniques, chain and segmental muscle stretching and active kinesiotherapy^(^ 13 ^-^ 15 ^)^. 

For data collection, three questionnaires were used: one that characterized the workers^(^ 8 ^)^ and approached the personal and occupational aspects of the subjects; the Working Stress Scale (WSS)^(^ 16 ^)^ to identify the presence of work-related stress and the Corlett Diagram (CD)^(^ 17 ^)^ to evaluate the presence, location and the intensity of musculoskeletal pain reported in the complaints, both adapted and validated for the use in Brazil.

The period of collection was from February to May 2010 and was divided into three stages: the first was the pre-test, which consisted of the application of the questionnaires to the subjects, after the completion of the Informed Consent Form; the second was the WE intervention, which was held twice a week for 15 minutes over a period of 10 weeks. This was held in the premises of the HEPI in a large and appropriate room for the practice of group activities, in the interim period of morning or afternoon, depending on the availability of the subjects to participate in the sessions.

The WE protocol adopted in this study was developed by the first author, using the following exercises and techniques: postural exercises, segmental stabilization and segmental and muscular chain stretching^(^ 13 ^-^ 15 ^)^. The segmental stretching were the most performed exercises, since they are typical of the WE and they occurred as follows: in the 1^st^ week, exercises for the cervical spine and neck were performed; in the 2^nd^ week, cervical and shoulder; 3^rd^ week, shoulder; 4^th ^week, forearm, wrist, hand; 5^th^ week, all segments of the upper limbs (cervical, shoulder, wrist and hand); 6^th^ week, spine; 7^th^ week, hip (flexor, extensor, adductor, abductor group); 8^th^ week, all segments of the lower limbs (posterior muscle chain) and active ankle and feet kinesiotherapy; and 9^th^ and 10^th^ weeks, combination of all cervical exercises, upper and lower limbs.

In conjunction to these, for the association of postural and segmental stabilization exercises and muscular chain stretching, in the 1^st^ week of intervention, the performance of retroversion and anterior pelvic till movements were taught, as well as self-enlargement of the spine associated with extended expiration to maintain the duration of the exercise. In addition to the segmental stretching, lying down exercises were associated in the 2^nd^, 3^rd^ and 4^th^ weeks; seated exercises in the 5^th^, 6^th^ and 7^th^ weeks and standing exercises in the 8^th^, 9^th^ and 10^th^ weeks.

The exercises were verbally explained and shown so that participants could have a better understanding of the movements and then execute them.

The third stage was the post-test, in which the subjects answered the WSS and the CD again. The questionnaires were applied on the day after the last day of intervention. Thus, there was a comparison between the pre and post-test scenarios.

After the collection, the data were entered into a *MS-Excel *spreadsheet using the double entry technique for validation. Later, they were exported and analyzed through the* Statistical Package for the Social Sciences *(SPSS) program, version 14.0. The descriptive statistics was used for presentation of the sample studied and for analysis of the data associated with the work-related stress and musculoskeletal pain variables concerning pre and post-test. In order to verify whether the scores obtained were significant, the *Kolmogorov-Smirnov *normality test was initially conducted and found non-normal data, being used for the non-parametric statistical analysis. Thus, the *Wilcoxon* Test was administered separately for the analysis of each one of the variables, work-related stress and musculoskeletal pain, with a significance level of α=0.05 for 95% reliability.

*Blox plots* were graphically used to represent the distribution of the scores of the work-related stress variables. In these, quartiles, minimum and maximum values and *outliers* are presented.

The implementation of this study was approved by the Research Ethics Committee of the HEPI under registration 0954/08.

## Results

Concerning the personal characteristics of the subjects, most of them were female (56.7%), married or in a de facto relationship (70%), with university degree (73.3%) and right handed (90%). Their average age was 41.7 (±8.79) and they performed physical activities 2.6 (±1.5) times a week in average. However, most of them performed physical activities only once a week (33.3%) followed by those who performed them four (23.3%) and three (20%) times a week. As for the occupational characteristics, they had an average of 40.13h (±0.7) working hours per week and all of them worked full time (morning and afternoon); most of them (73.3%) reported not to work overtime and 13.3% had another job.

**Figure 1:**
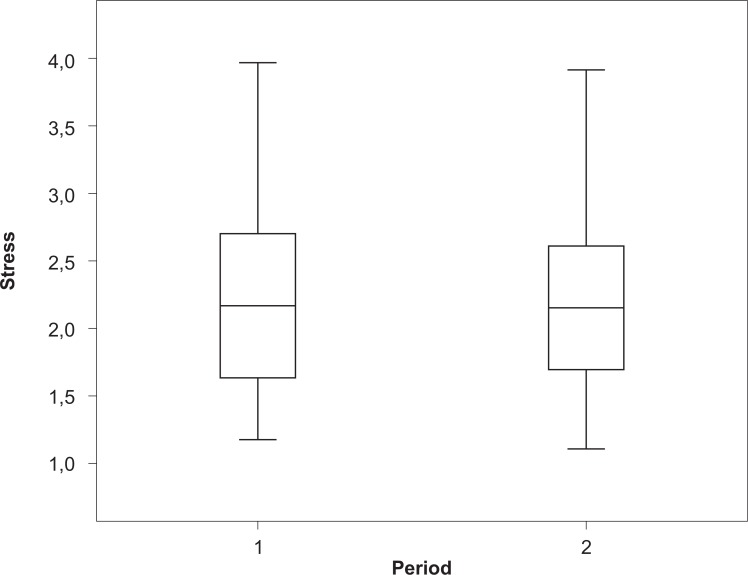
Box plots related to the scores of work-related stress before (1) and after (2) the performance of Workplace Gymnastics by administrative employees working in a Higher Education Public Institution. Ribeirao Preto, SP, Brazil, 2010 (N=30)

The average score concerning the presence of work-related stress among these workers was 2.3(±0.7) in the pre-test and 2.2(±0.7) in the post-test, and the minimum and maximum scores are shown in Figure 1. In relation to the significance of the change of complaints about work-related stress, the *Wilcoxon* test showed no statistically significant change between the pre and the post-test (p=0.150).

In terms of symptoms of musculoskeletal pain and discomfort among the employees before and after the WE, the mentioned data were presented by segments of the spine (SP), upper limb (UL) and lower limb (LL).

Concerning the SP, it was found that both the symptoms of pain and its intensity in all the segments assessed were reduced. The *Wilcoxon* test showed a significant reduction of the symptoms in all assessed segments, such as: neck (p=0.007), cervical (p=0.02), upper back (p=0.02), mid back (p=0.012) and lower back (p=0.032) (Table 1).

**Table 1:** Median values and statistical significance obtained through the Wilcoxon test concerning the reduction of pain in the segments of the spine, upper limb and lower limb before and after the Workplace Exercise practice by administrative employees working in a Higher Education Public Institution. Ribeirao Preto, SP, Brazil, 2010 (N=30).

Body segment		Median values *Wilcoxon* Test		Significance
		Pre-test	Post-test	
Spine				
	Neck	2.0	1.0	0.007*
	Cervical	2.0	1.0	0.02^†^
	Back (upper)	2.0	1.0	0.02^†^
	Back (mid)	1.0	1.0	0.012^†^
	Back (lower)	2.0	2.0	0.032^†^
	Hip	1.0	1.0	0.66
Upper limb				
	Right Shoulder	1.0	1.0	0.305
	Left Shoulder	1.0	1.0	0.873
	Right Arm	1.0	1.0	0.121
	Left Arm	1.0	1.0	0.785
	Right Elbow	1.0	1.0	0.785
	Left Elbow	1.0	1.0	1.000
	Right Forearm	1.0	1.0	0.317
	Left Forearm	1.0	1.0	0.589
	Right Wrist	1.0	1.0	0.101
	Left Wrist	1.0	1.0	0.417
	Right Hand	1.0	1.0	0.201
	Left Hand	1.0	1.0	0.453
Lower limb				
	Right Thigh	1.0	1.0	0.038^†^
	Left Thigh	1.0	1.0	0.063
	Right Knee	1.0	1.0	0.429
	Left Knee	1.0	1.0	0.112
	Right Leg	1.0	1.0	0.71
	Left Leg	1.0	1.0	0.047^†^
	Right Ankle	1.0	1.0	0.009^†^
	Left Ankle	1.0	1.0	0.145
	Right Foot	1.0	1.0	0.026^†^
	Left Foot	1.0	1.0	0.013^†^

p : Wilcoxon test

* p<p<0.005

Descriptively, there is reduction of "moderate" and "strong" pain in the neck (from 26.7% to 6.7%), cervical (from 16.7% to 0%) and upper (from 40% to 3.3%), mid (from 26.7% to 3.3%) and lower back (from 33.3% to 10%). In addition to the reduction of pain intensity, there were no complaints in all these segments of the spine at the post-test, which suggests that the WE could stop pain symptoms in some employees.

In relation to the UL, there was no statistically significant pain reduction in the segments assessed (Table 1).

Concerning the LL, some segments showed statistically significant pain reduction, such as: right thigh (p=0.038), left leg (p=0.047), right ankle (p=0.009), right foot (p=0.026) and left foot (p=0.013) (Table 1).

There was a reduction of "moderate" and "strong" pain intensity in the segments right thigh and right and left legs (from 10% to 0), right ankle (from 16.7% to 6.7%) and right foot (from 20% to 13.3%), as well as in the item "unbearable" that was reported only in ankles and feet, having this complaint been ended in relation to both segments.

## Discussion

The personal and occupational profiles of the research participants were similar to those of other researches in which state public staff participated in WE programs^(^ 18 ^-^ 19 ^)^.

The aim of identifying whether there is stress among employees before and after the WE, according to determinants of the framework used^(^ 16 ^)^ showed that there is mild work-related stress among the people assessed, with an average of 2.27 in the pre-test. It is believed that the average score of the WSS is around 2 and 2.5; higher scores than these indicate high level of stress, and lower scores show a lack of it. In the post-test, the average score obtained was 2.16, also showing low level of stress, which did not have a statistically significant reduction (p=0.150) after the proposed intervention.

Work-related stressful events are often related to the work organization, such as pressure for productivity, retaliation, unfavorable conditions to work safety, unavailability of training and guidance, abusive relationship between supervisors and subordinates, lack of control over tasks and work-rest cycles inconsistent with biological limits^(^ 4 ^-^ 5 ^)^. This situation encourages short and long term responses which increase the possibility of developing different work-related illnesses that affect the physical and psychological health^(^ 5 ^,^ 20 ^)^ and can lead to human and economic losses associated with work-related stress, resulting in the need for physical and/or psychological interventions in order to manage, prevent or control them, and to promote a healthy range of strategies to cope with the stress^(^ 21 ^)^.

In the present study, the intervention proposed was the WE, which is focused on the individual and which, according to the results obtained, was not able to reduce the complaints of work-related stress by these subjects.

As for the complaints about musculoskeletal pain, there was a pain reduction in all of the segments assessed, which is more significant in parts of the SP and in the LL, except knees. In relation to the UL, all the segments assessed showed to have had a reduction in the complaints of pain; however, these were not statistically significant. Other researchers that used the WE to reduce musculoskeletal pain or stress had similar results to those showed in this study^(^ 7 ^-^ 8 ^,^ 18 ^,^ 22 ^-^ 24 ^)^. However, since a WE protocol should be designed specifically to each workplace sector or environment where it will be applied because every workplace reality will demand certain kinds of exercises and therapeutic procedures, it is difficult to compare it with that from other results, even though some studies have assessed the same variables assessed in this investigation, because the exercises, frequency, duration and intensity used were not the same in all of the studies. Therefore, a comparison is made with the WE as a physiotherapy technique of work intervention, but the comparison and discussion based on the exercises covered and performed by the subjects is not possible to be made.

One explanation for the significant pain reduction in the SP showed in this study may be the use of lower back segmental stabilization techniques^(^ 22 ^,^ 24 ^)^ and the *Isostretching* ^(^ 13 ^)^ performed in the sessions, which are more focused on the stretching and strengthening of muscles that support this area. It is likely that the use of these two techniques in the protocol of exercises performed in the WE sessions cause such pain reductions, especially the spine, thoracic cervical, thoracic and lower back.

The effectiveness of segmental stabilization as treatment for back pain is proven, and it is less harmful due to its performance in a neutral position. Without the correct activation of the deep stabilizers of the torso, the recurrence of pain in the spine is noted more frequently. Therefore, exercises of synchronized, subtle and specific isometric contractions are proposed, which act directly on the relief of pain by increasing the stability of the spinal segment^(^ 22 ^-^ 24 ^)^.

The method *Isostretching* is also consistent with these concepts, making most muscular groups to work concentrically (shortening by contraction) and eccentric (lengthening by controlled relaxation), without application of power overload and impact on the joints, allowing the optimization of the muscle activity, addition of power and mobility and therefore adjust the natural curves of the body^(^ 13 ^)^.

Based on this clinical, social and occupational importance of the functional alterations likely to appear in the spine, these concepts were the basis for the development and choice of exercises that composed the WE protocol that was structured for this study, which certainly provided a positive effect in the lives of the participants, because they were able to experience a significant pain reduction in the segments of the spine assessed.

Concerning the joint analysis of the work-related stress and musculoskeletal pain, the literature shows that both of them are interrelated, since the first one may ultimately influence and support the second one due to a combination of casual or correlational relationship between the physical and psychosocial needs^(^ 1 ^-^ 5 ^)^. However, this somatic and associative issue between stress and pain was not completely proved in this investigation because, although a significant reduction of musculoskeletal pain was verified, the same result was not verified in relation to work-related stress and this might have been a result of the number of participants.

## Conclusion

The compensatory WE program carried out caused, to administrative employees, a reduction of musculoskeletal pain in most of the body segments assessed. Concerning the spine, there was pain reduction in all segments, reduction of pain intensity and lack of symptoms in all segments in the post-test, suggesting that the WE could stop the pain of the participants, and that the decrease of symptoms in the neck, cervical, upper, mid and lower back was statistically significant.

Concerning the UL, there was no statistically significant pain reduction in the assessed segments. As for the LL, there was a statistically significant pain reduction in the right thigh, left leg, right ankle and feet.

The WE protocol used in this study did not significantly reduce the complaints of work-related stress and the average levels remained the same before and after the test.

It is noteworthy that other ways and instruments used to measure work-related stress focused on workers, involving a larger number of participants, can be carried out, with focus on the physical and psychological issues of the subject, and not only on the organizational area in which this subject is, in an associative manner and seeking to identify a more complex scope of this context. It is suggested that this study is reapplied to a sample with a larger number of subjects, which was not contemplated in the present study.
